# The Immediate Aesthetic and Functional Restoration of Maxillary Incisors Compromised by Periodontitis Using Short Implants with Single Crown Restorations: A Minimally Invasive Approach and Five-Year Follow-Up

**DOI:** 10.1155/2015/716380

**Published:** 2015-11-15

**Authors:** Mauro Marincola, Giorgio Lombardo, Jacopo Pighi, Giovanni Corrocher, Anna Mascellaro, Jeffrey Lehrberg, Pier Francesco Nocini

**Affiliations:** ^1^Universidad de Cartagena, Avenida del Consulado, Calle No. 30, No. 48-152, Cartagena, Bolívar, Colombia; ^2^Clinic of Dentistry and Maxillofacial Surgery, University of Verona, Piazzale Ludovico Antonio Scuro 10, 37100 Verona, Italy; ^3^Department of Biomaterials, Implant Dentistry Centre, 501 Arborway, Jamaica Plain, Boston, MA 02130, USA

## Abstract

The functional and aesthetic restoration of teeth compromised due to aggressive periodontitis presents numerous challenges for the clinician. Horizontal bone loss and soft tissue destruction resulting from periodontitis can impede implant placement and the regeneration of an aesthetically pleasing gingival smile line, often requiring bone augmentation and mucogingival surgery, respectively. Conservative approaches to the treatment of aggressive periodontitis (i.e., treatments that use minimally invasive tools and techniques) have been purported to yield positive outcomes. Here, we report on the treatment and five-year follow-up of patient suffering from aggressive periodontitis using a minimally invasive surgical technique and implant system. By using the methods described herein, we were able to achieve the immediate aesthetic and functional restoration of the maxillary incisors in a case that would otherwise require bone augmentation and extensive mucogingival surgery. This technique represents a conservative and efficacious alternative to the aesthetic and functional replacement of teeth compromised due to aggressive periodontitis.

## 1. Introduction

The assortment of maladies that constitute the broadly defined periodontal disease runs the gamut from relatively benign to life threatening [1, 2]. The aetiology of periodontal disease (and its subsequent severity) has a number of factors, including deleterious bacteria in the oral environment, genetic predisposition, and host inflammatory and immune responses [2–4]. One of the more severe forms of periodontal disease—aggressive periodontitis—is characterized by destruction of the periodontal ligament, recession of the gingival smile line, and horizontal resorption of the alveolar bone [3, 5]. Depending on a number of variables, including the progression of the disease and the compliance of the patient, aggressive periodontitis can be managed through nonsurgical methods [3, 6]. However, in cases of advanced aggressive periodontitis, surgical therapy may be indicated [6].

In cases of aggressive periodontitis where the extent of the disease necessitates surgical intervention, procedures that take a minimally invasive approach have been shown to be advantageous in regard to tooth preservation [6, 7]. Nevertheless, if treatment of aggressive periodontitis is delayed for extended periods, then salvage of the affected teeth may be unattainable; and removal of compromised teeth is advised [4]. Dental implants have become a popular method to restore the aesthetics and functionality of teeth lost to aggressive periodontitis [8, 9]. However, implant placement in sites lost to periodontitis (i.e., locations characterized by horizontal bone resorption) typically requires concomitant bone augmentation procedures, a modality that increases the length of the healing and cost of the procedure and has unpredictable aesthetic outcomes [10, 11]. Here we report on a minimally invasive surgical technique using a short implant system that allowed us to fully restore the maxillary anterior incisors in a 37-year-old female patient who possessed extensive horizontal bone loss due to aggressive periodontitis. Using this technique and implant system we were able to functionally and aesthetically restore the compromised anterior teeth, without the use of bone augmentation procedures.

## 2. Case Presentation

**Figure 1:**
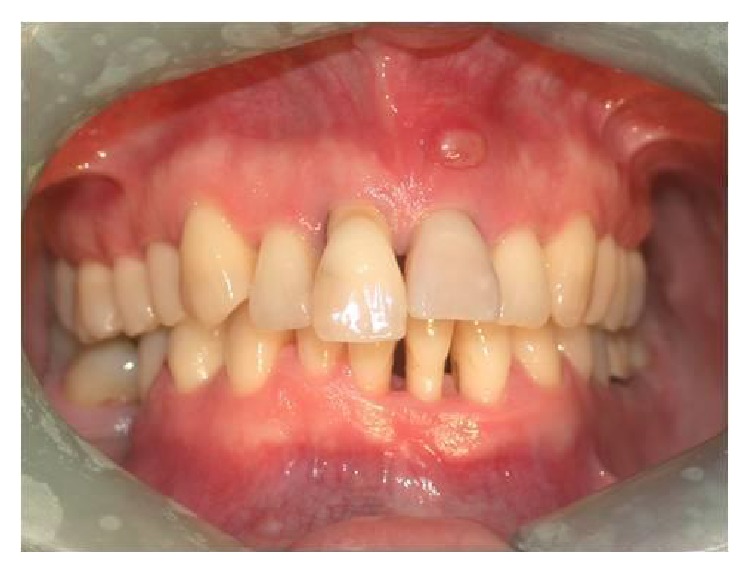
Extent of the patient's periodontitis upon presentation.

**Figure 2:**
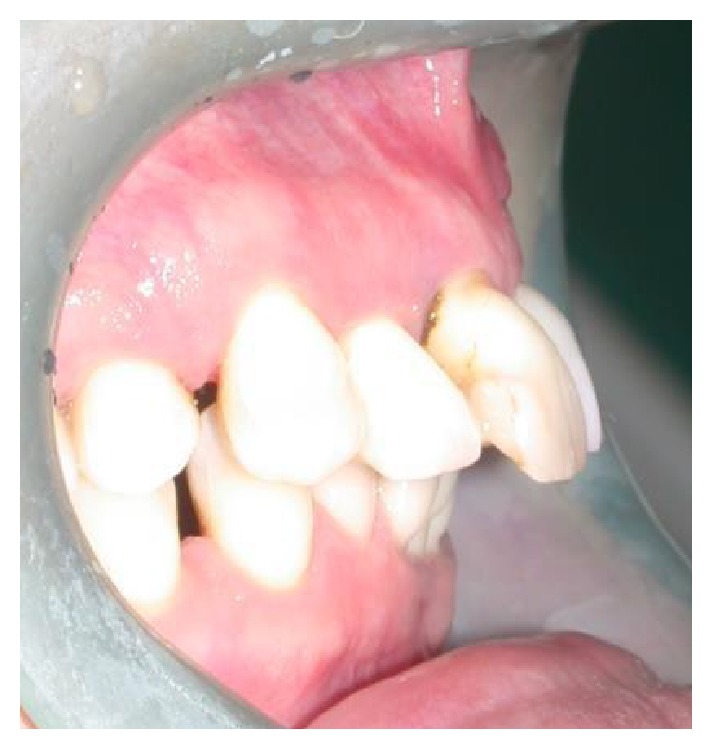
Right side view of patient upon presentation.

**Figure 3:**
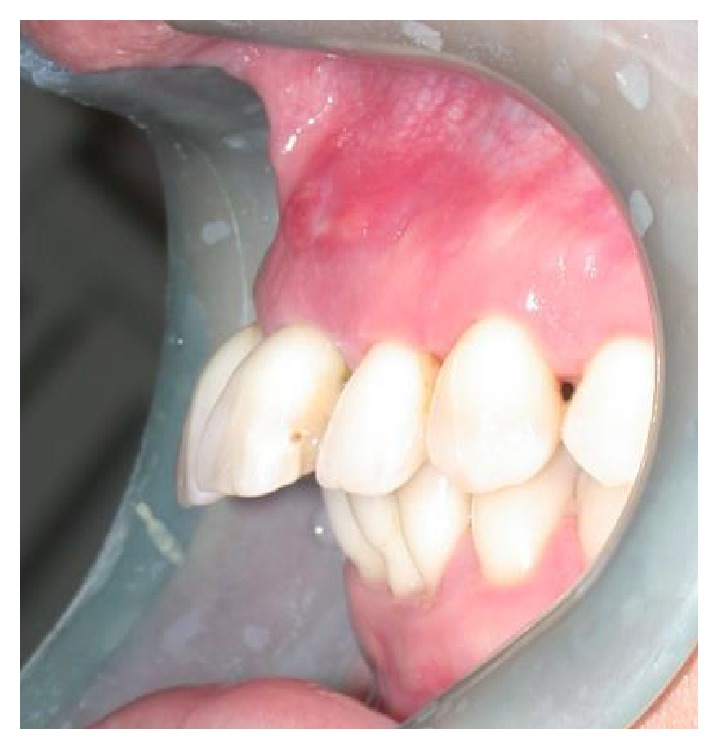
Left side view of patient upon presentation.

**Figure 4:**
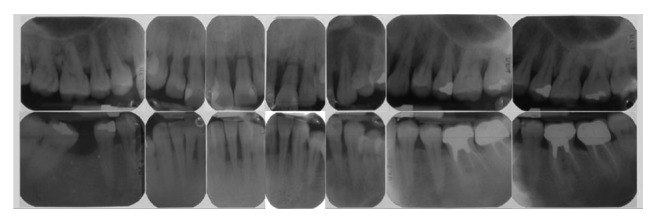
Intraoral periapical radiographs showing horizontal bone loss at all four maxillary incisors.

**Figure 5:**
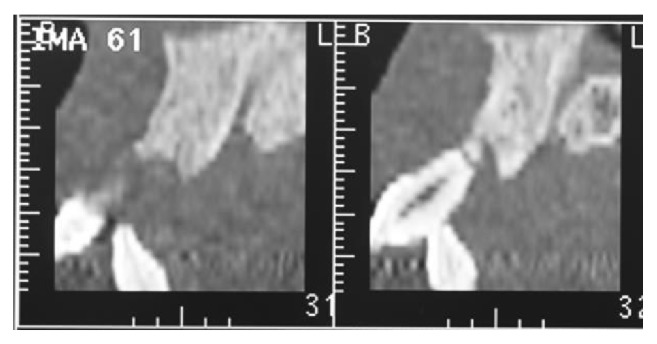
CT scans of patient upon presentation showing resorption of buccal and palatal bone adjacent to maxillary incisors.

A 37-year-old female patient reported to us expressing concern over the mobility of her teeth, the presence of a recurrent fistula, and overall displeasure with the height of her gingival smile line (Figure 1). Upon clinical examination, it was determined that the maxillary incisors (i.e., teeth numbers 7, 8, 9, and 10) were compromised and that horizontal bone resorption had occurred as a result of aggressive periodontitis. The right central incisor was extruded and dislocated due to secondary occlusal trauma, and the left central incisor possessed a horizontal root fracture (Figures 2 and 3). Intraoral periapical radiographs revealed horizontal bone defects in all four of the maxillary incisors (Figure 4). Severe resorption of the alveolar ridge in the premaxillary area, along with complete resorption of the buccal and palatal bones adjacent to the roots of maxillary incisors, was also observed (Figure 5).

After apprising the patient of her situation, we offered a number of viable treatment options; however, the patient has adamantly opposed many of them. Because the patient did not wish to use dentures and wanted to retain the ability to floss between prosthetic teeth, we were restricted from implementing a number of conventional treatment options. Despite being constrained by both the extent of patient's periodontitis and her aforementioned wishes, we nevertheless outlined a treatment plan that conformed to the patient's desires and would restore the aesthetics and functionality of the compromised teeth. In agreement with the patient, it was decided that the compromised incisors be extracted and replaced with four short locking-taper implants using a minimally invasive surgical technique which would not require flap raising or bone grafting procedures.

**Figure 6:**
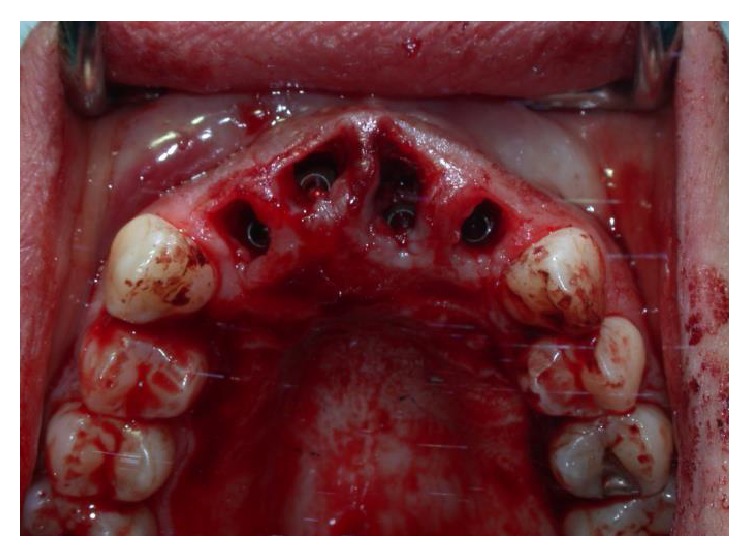
Postsurgical placement of Bicon implants. The implants lie 2-3 mm below the alveolar crest.

**Figure 7:**
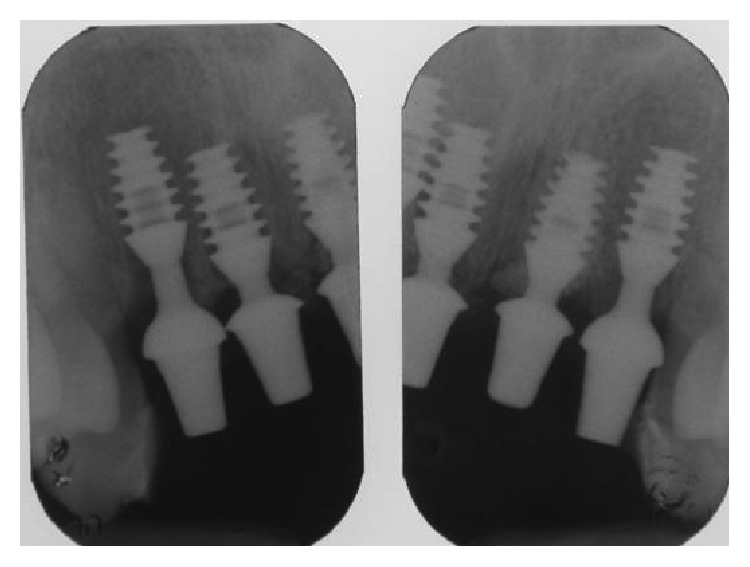
Postsurgical X-ray showing implants after surgery with provisional abutments inserted.

**Figure 8:**
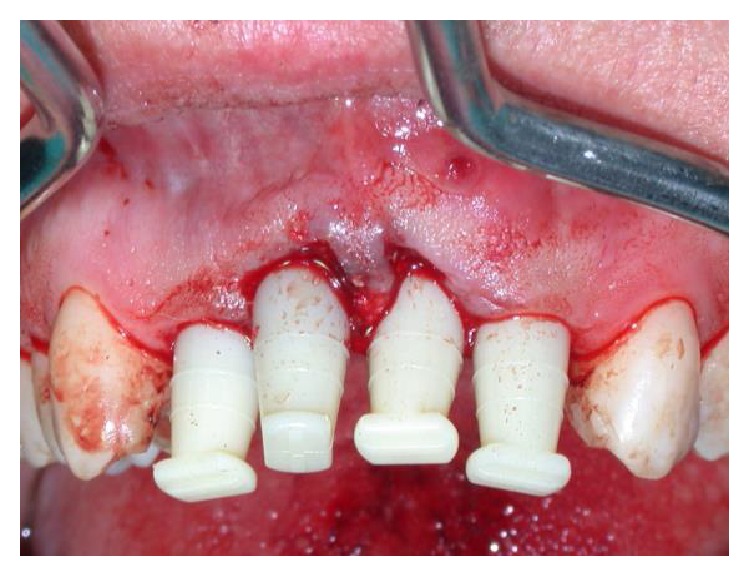
Preformed shouldered abutments with polycarbonate snap-on sleeves.

**Figure 9:**
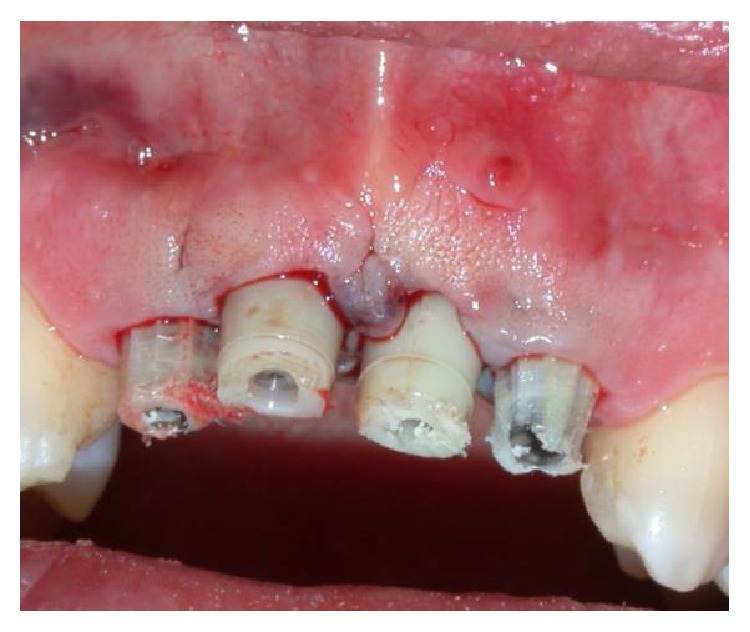
Polycarbonate snap-on sleeves adapted to receive temporary bridge.

The patient was treated with a local anaesthetic prior to extraction (4% Articaine with 1 : 100,00 adrenaline, Ubistesin R; 3M ESPE). Care was taken to extract the teeth with minimum trauma so as not to damage the buccal or palatal bone plates. The compromised teeth were luxated and extracted while avoiding lateral movement. Following extraction of the affected teeth, the implant sites were prepared using a 2.0 mm diameter pilot drill and a 2.5 mm drill on an 18 : 1 hand piece at 1000 RPM with constant irrigation (2.0 mm Standard Pilot Drill, Bicon LLC, Boston, MA). Using a 400 : 1 hand piece at 50 RPM, the osteotomies were produced by the sequential use of 2.5, 3.0, and 3.5 mm reamers (Latch Reamers, Bicon LLC, Boston, MA), followed by hand reaming with a 4.0 mm reamer (diameter of implant). Bone obtained from the reamers was stored in a silicone dappen dish for later grafting. The osteotomies were generated to a final depth that would result in the implant shoulders lying 2.5 mm below the alveolar crest. Four endosseous root-form short implants (4.0 × 8.0 mm MAX 2.5 Implants, part #260-340-008, Bicon LLC, Boston, MA) were then inserted using the manufacturers inserter and further tapped in using a seating tip (Figure 6). The implant placed in position 8 was inserted in more vestibular position in order to accommodate its size and prevent interference between prospective prosthetics (i.e., prosthetic on the lateral incisor). Bone harvested during reaming, along with tricalcium phosphate (SynthoGraft Pure Phase Beta-Tricalcium Phosphate, part #260-400-150 Bicon LLC, Boston, MA), was applied to the shoulder of the implant; healing plugs were used to avoid the deposition of bone graft particles inside the implant well. The healing plugs were then replaced by preformed shouldered parallel abutments (Universal Stealth-Shouldered Abutment, Bicon LLC, Boston, MA) upon which polycarbonate snap-on sleeves (Temporization Sleeves, Bicon LLC, Boston, MA) were adapted to receive an immediate temporary restoration (Figures 7, 8, and 9).

**Figure 10:**
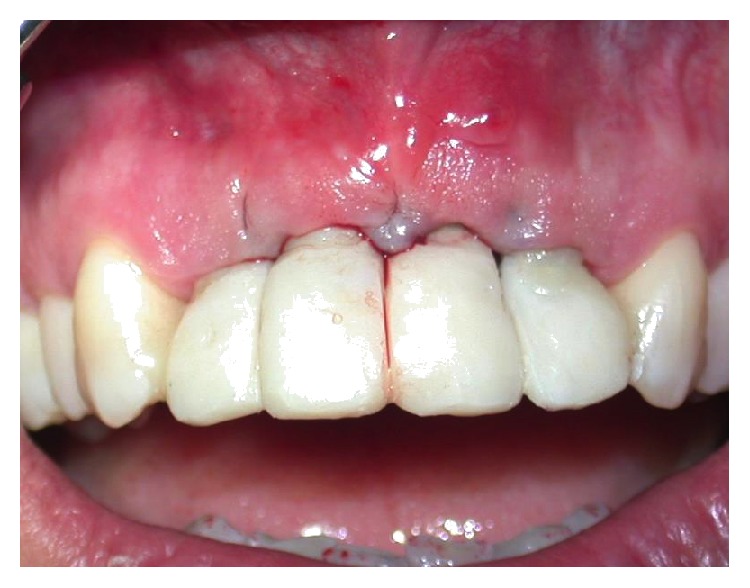
The immediate temporary nonfunctional bridge after seating and adjustment.

**Figure 11:**
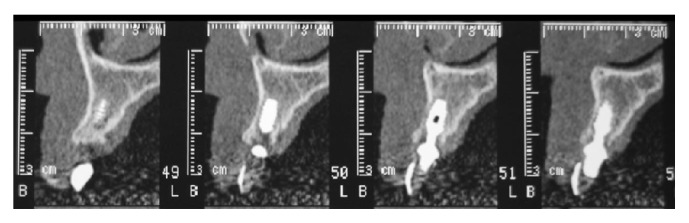
CT scans three weeks after surgery. Note the absence of vestibular bone dehiscence.

**Figure 12:**
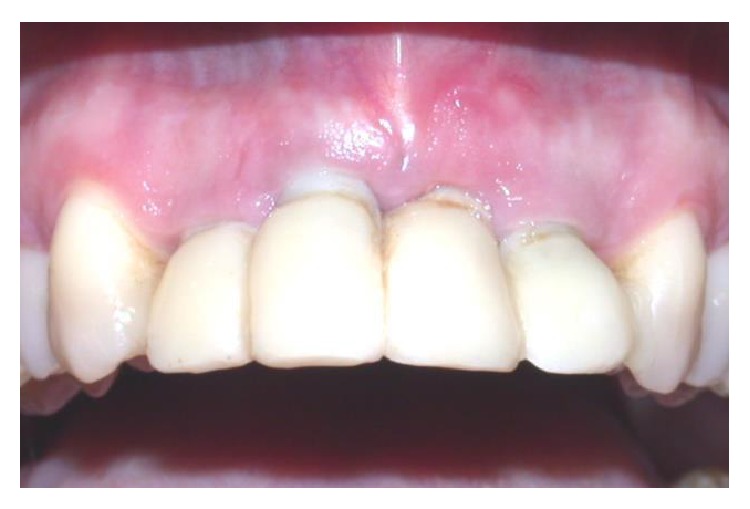
Slight gingival recession observed at tooth number 8, one week after surgery.

The immediate temporary restoration consisted of a nonfunctional temporary bridge, which was seated and adjusted to clear centric and eccentric contacts and to support the papillae without encroachment (Figure 10). The implementation of a “snap-on” system between the abutment and emergence sleeve precluded the use of cementation, which in turn abrogated the potential for soft tissue irritation. Postoperative care included 2 grams of daily oral antibiotics for 6 days (Augmentin, GlaxoSmithKline, Verona, Italy). Additionally, the patient was given detailed postoperative instructions about analgesic therapy and oral hygiene, along with a 0.12% chlorhexidine mouth rinse to be administered 3 times a day for 7 days (GUM PAROEX Chlorhexidine Gluconate Oral Rinse 0,12% CHX + 0,05% CPC, Sunstar Suisse S.A., Etoy, Switzerland). Three weeks after surgery, CT scans confirmed implant position and were absent for signs of vestibular bone dehiscence (Figure 11); furthermore, the patient exhibited good wound healing (with the exception of slight gingival recession at tooth number 8—see Discussion) (Figure 12).

**Figure 13:**
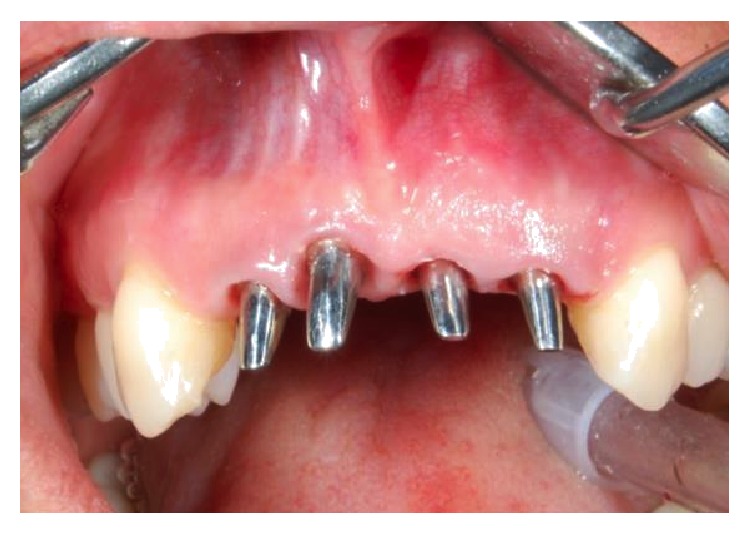
Soft tissue prior to delivering.

**Figure 14:**
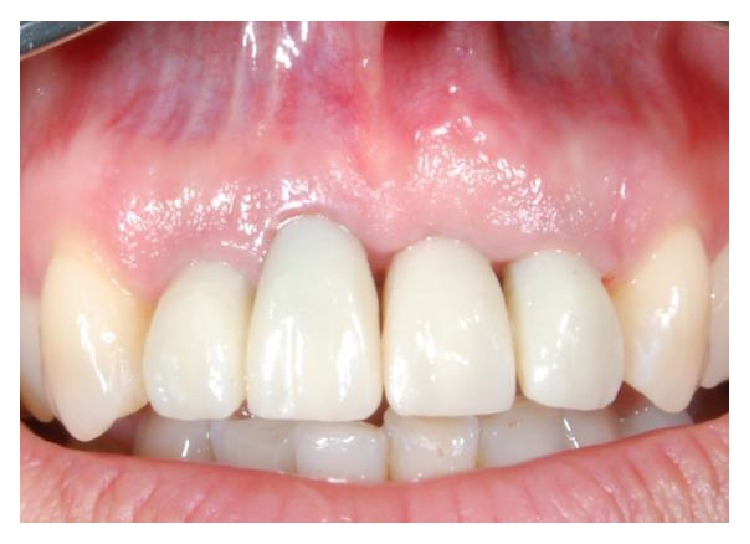
The temporary bridge, three months after surgery.

**Figure 15:**
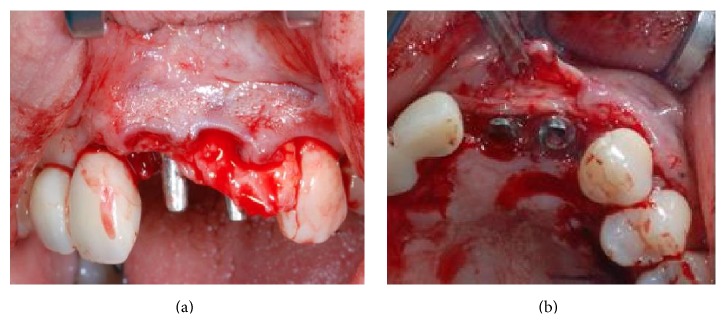
Tissue graft being positioned at implant site #8.

**Figure 16:**
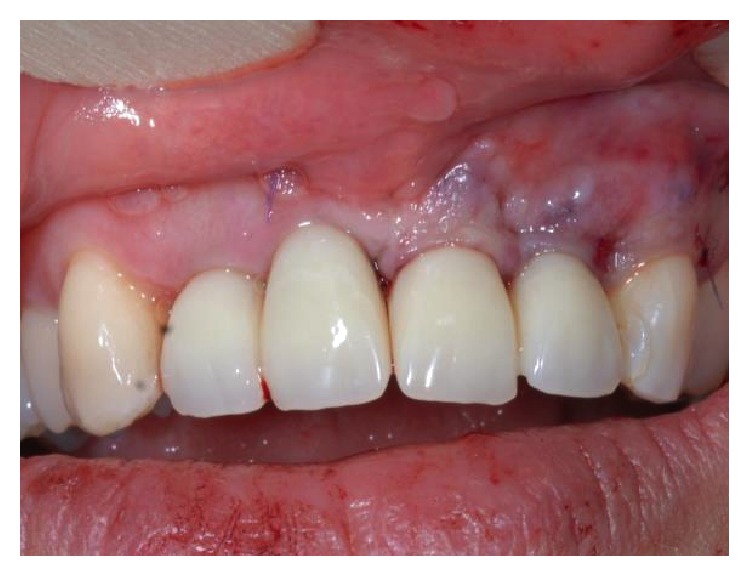
Image showing soft tissues after tissue grafting.

**Figure 17:**
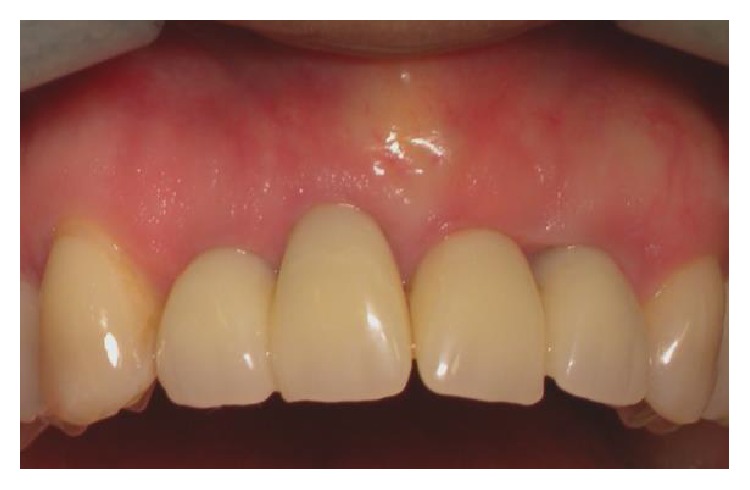
Soft tissue three months after tissue graft.

Three months following the procedure, the temporary bridge was then replaced by two temporary prostheses (supported by implants in positions #7 and #8 and in positions #9 and #10) to guide the regrowth of the gingival contours (Figures 13 and 14). Four months following the placement of the temporary prostheses, we performed two connective tissue grafts to thicken peri-implant soft tissues promoting a more natural and aesthetic emergence profile. Connective tissue for the first graft was harvested from the left palatal mucosa and grafted to the implants in positions #7-8. Then, after a two-month healing period, tissue was harvested from the right palatal mucosa and grafted to the implants in positions #9-10 (Figures 15 and 16). Both tissue grafts were harvested without epithelial layer using the trap door technique. Three months after the second tissue graft procedure, the patient had healed sufficiently enough to allow the placement of the final restorations (Figure 17).

Lateral/anterior protrusion of the definitive restorations was achieved using canine guidance: in this way, mutually protected occlusion prevented contact between incisors during all mandibular eccentric movements, and incisors came into contact with their antagonists only during maximum intercuspation.

**Figure 18:**
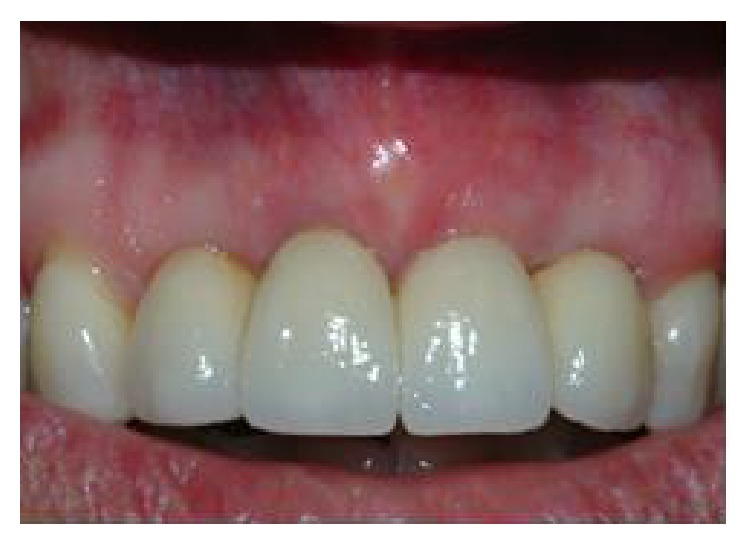
Image depicting the final restorations after delivery.

To orient the seating of the final abutment, a jig was fabricated and utilized to aid in correct positioning. A direct impression was then taken using a polyether impression material (Impregum Penta, 3M ESPE, St. Paul, MN). We then prepared a stone cast using type IV extra-hard dental stone, from which the definitive abutment could be individually modified. Finally, four zirconia crowns were fabricated and cemented on the abutments using extraoral cement (RelyX Unicem, 3M ESPE, St. Paul, MN). The abutment and crown were then tapped through the long axis of the post into the implant well using a 250 g mallet (Figure 18).

## 3. Results

**Figure 19:**
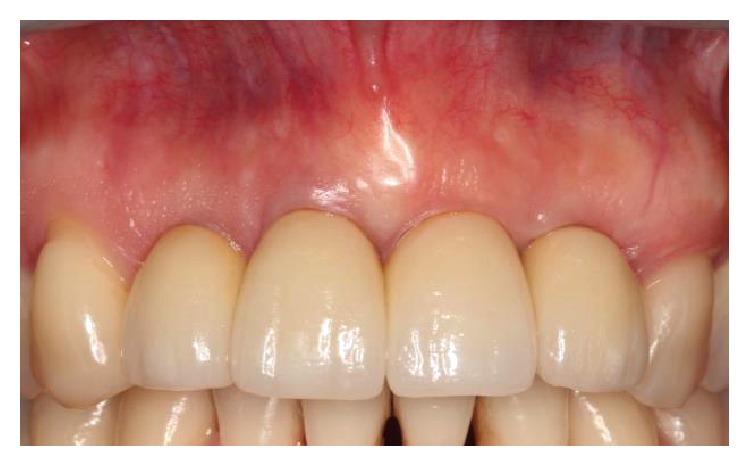
Front view of restoration at five-year follow-up.

**Figure 20:**
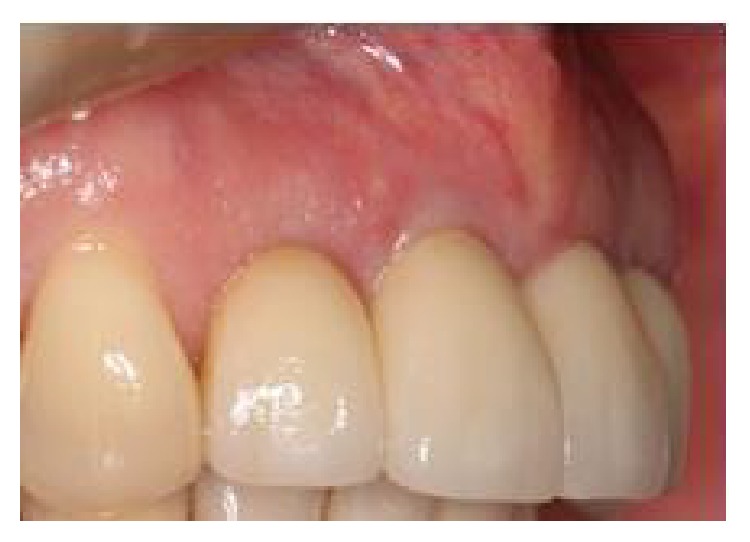
Close-up of tissue graft site and restoration at five-year follow-up.

**Figure 21:**
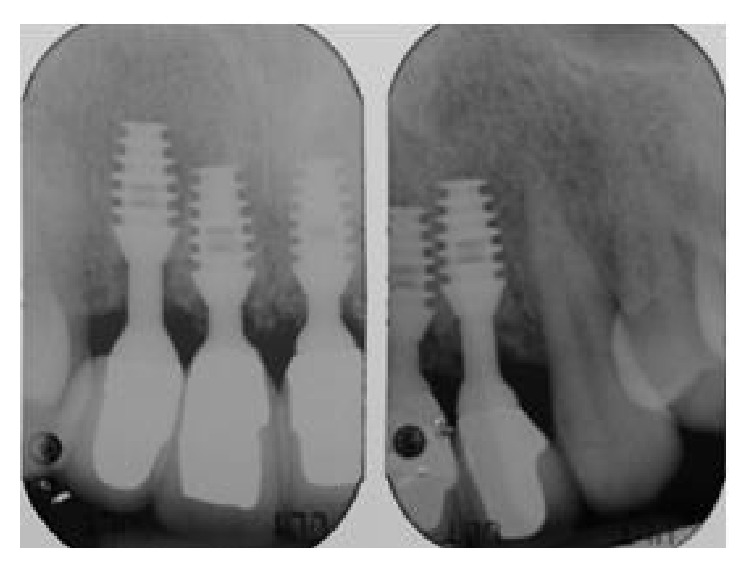
Radiographs at five-year follow-up showing interproximal bone and bone at implant shoulder.

**Figure 22:**
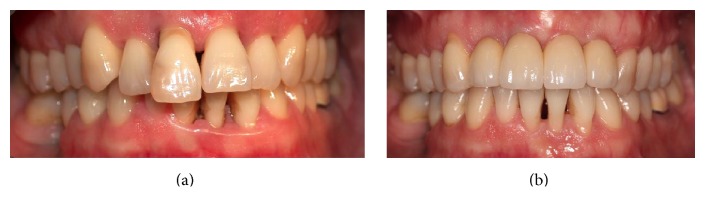
Before and after image depicting the patient upon presentation (a) and the patient at five-year follow-up (b).

Five years after placement of the final restorations, the patient was pleased to report that she found the gingival margins aesthetically pleasing and found no functional difference between the implants and her natural teeth. Over the five-year period since the surgery, the gingival margins have remained stable, and the implants have remained perfectly integrated. The peri-implant tissues surrounding all four implants lack any visible signs of inflammation or plaque and do not produce bleeding upon probing (Figures 19 and 20). Intraoral radiographs confirmed the long-term stability of the alveolar crest around the sloping shoulders of the implant necks and the presence of interproximal bone (Figure 21). The patient concluded that she was fully satisfied with both the aesthetic and functional results of the procedure (Figure 22).

## 4. Discussion

Restoration of missing teeth in the maxillary aesthetic zone presents several clinical challenges even when performed under ideal conditions; bone loss due to periodontitis serves to exacerbate these challenges. Periodontitis induced horizontal bone loss, and the morphology that results restricts the number of treatment options available to clinician and patient alike [12, 13]. As illustrated in the present case, many of the conventional treatments available for the restoration of lost teeth in patients suffering from aggressive periodontitis (dentures, large span bridgework, etc.) are unpopular; moreover, they have a greater negative impact on the psychological well-being of their recipients when compared to dental implants [8, 9]. And while dental implants offer an alternative to removable prostheses, their usage in the anterior maxilla is encumbered by morphological aspects of the bone, especially so in patients with periodontitis [3, 6, 12].

Bone resorption owing to periodontitis in the aesthetic zone makes aesthetically pleasing and convincing restorations difficult by interfering with implant placement and preventing the regeneration of a natural looking gingival smile line [3, 6, 12–14]. This report describes the restoration of anterior maxillary incisors in the aesthetic zone compromised by aggressive periodontitis using a minimally invasive technique and short implants. Due to the patient's periodontitis and desired final outcome, treatment options were restricted. Patient preference further prohibited the use of removable prostheses or fixed bridges, and patient morphology and horizontal bone loss limited the number of possible implant system choices. To overcome the challenges this case presented, we decided to use a minimally invasive surgical technique in concert with the postextractive placement of short implants: a modality that satisfied both the functional and aesthetic requirements, along with the patient's wishes.

We were initially concerned that the close proximity of the implants (both to each other and to the adjacent teeth) might negatively impact the outcome of the procedure; therefore, we chose an implant system that was particularly suited for the conditions described herein [15–17]. The small size of the selected implant system allowed the placement of four individual implants with corresponding crowns in regions where aggressive periodontitis had caused excessive horizontal bone loss, a feat which would be impossible using larger implant systems. The unique macrogeometry of the implant system (i.e., sloping shoulder) afforded space for interproximal bone growth, which consequently supported the aesthetically pleasing interdental papillae that developed [18, 19]. Furthermore, in comparison to an implant-supported bridge, the use of four individual crowns will facilitate greater hygienic maintenance in the long term and—perhaps more importantly—was in compliance with the patient's wishes.

We recognized that choosing not to splint the implants—along with choosing to use short implants in the first place—might affect the long-term success of this procedure, as it has been demonstrated that the crown height can act as a vertical cantilever, and an angled prosthetic load magnifies the force applied on implants (i.e., overloading). To offset the potential for overloading, we minimized the nonaxial forces applied to single crown implants by maintaining the preexisting canine guide. Additionally, the plateau design of the implants used reports a surface area 30% higher than other screw form implants of similar length, further reducing the potential for overloading [20].

However, due to this peri-implant design, probing depths using the periodontal probe must be taken into consideration carefully, because the implants hemispherical base should negate the vertical use of a periodontal probe. Nonetheless, the absence of bleeding on probing along with the absence of plaque around prosthetic crowns is definitely a positive sign supporting the health of peri-implant tissues.

While the short implant system imparted much greater flexibility in regard to potential treatment options, we nevertheless were required to perform tissue grafts. Destruction of connective tissue and concomitant gingival recession is a hallmark of periodontitis and is observed in implant procedures in general [3, 12]. However, in this particular case, this was expected due the vestibular placement of the implant in position 8. Nevertheless, the sloping shoulder of the implant system we implemented allowed for increased interproximal soft tissue growth and vascularization and yielded better outcomes where mucogingival surgery was concerned.

To the best of our knowledge, this is the first report concerning the immediate placement and loading of four short implants into fresh maxillary alveolar sockets to restore the incisors group single crown restorations. Based on the positive functional and aesthetic outcomes observed in this case report, we conclude that a minimally invasive, aesthetically pleasing, and functionally stable restoration of anterior maxillary incisors can be achieved using an implant design with platform switching at implant level and at abutment level in regions of bone compromised by periodontitis.
